# Cluster analysis of protein array results via similarity of Gene Ontology annotation

**DOI:** 10.1186/1471-2105-7-338

**Published:** 2006-07-12

**Authors:** Cheryl Wolting, C Jane McGlade, David Tritchler

**Affiliations:** 1 Department of Medical Biophysics, University of Toronto, Toronto, Canada; 2 Arthur and Sonia Labatt Brain Tumour Research Centre, Department of Cell Biology, Hospital for Sick Children, 555 University Avenue, Toronto M5G 1X8, Canada; 3 Ontario Cancer Institute, Princess Margaret Hospital, 610 University Avenue, Toronto M5G 2M9, Canada

## Abstract

**Background:**

With the advent of high-throughput proteomic experiments such as arrays of purified proteins comes the need to analyse sets of proteins as an ensemble, as opposed to the traditional one-protein-at-a-time approach. Although there are several publicly available tools that facilitate the analysis of protein sets, they do not display integrated results in an easily-interpreted image or do not allow the user to specify the proteins to be analysed.

**Results:**

We developed a novel computational approach to analyse the annotation of sets of molecules. As proof of principle, we analysed two sets of proteins identified in published protein array screens. The distance between any two proteins was measured as the graph similarity between their Gene Ontology (GO) annotations. These distances were then clustered to highlight subsets of proteins sharing related GO annotation. In the first set of proteins found to bind small molecule inhibitors of rapamycin, we identified three subsets containing four or five proteins each that may help to elucidate how rapamycin affects cell growth whereas the original authors chose only one novel protein from the array results for further study. In a set of phosphoinositide-binding proteins, we identified subsets of proteins associated with different intracellular structures that were not highlighted by the analysis performed in the original publication.

**Conclusion:**

By determining the distances between annotations, our methodology reveals trends and enrichment of proteins of particular functions within high-throughput datasets at a higher sensitivity than perusal of end-point annotations. In an era of increasingly complex datasets, such tools will help in the formulation of new, testable hypotheses from high-throughput experimental data.

## Background

The advent of high-throughput (HTP) investigation of proteins using proteomic methodologies has created a need for new approaches in bioinformatic analysis of experimental results. Most publicly available databases display information about proteins one record at a time [1-5]. This is useful in the case where the number of proteins of interest is small. However, a set of proteins identified in a typical proteomic experiment may contain tens, hundreds or even thousands of proteins to analyse [6-9], at which point it is no longer feasible to collect information one protein at a time. In addition, there may be patterns or subsets of interest that exist within the set of proteins that are not obvious if the proteins are analysed one at a time. Thus, analysis of data generated in HTP experiments requires tools that allow the integrated analysis and interpretation of a collection of proteins.

Several freely available tools facilitate analysis of sets of proteins or gene products. PANDORA clusters sets of proteins according to shared annotation and displays the results as a directed acyclic graph (DAG) [10]. Many types of annotation are incorporated, including Gene Ontology (GO) annotation [11]. PANDORA provides sets of proteins or allows the user to input a list of proteins of interest. SGD [1,2] provides the yeast community with the tools GO Term Finder, GO Slim Mapper and GO Annotation Summary for the analysis of a protein and all its interactors as found in SGD. WebGestalt permits the user to input interesting sets of genes and identify up to 20 types of annotation to be employed [12]. The sets can then be visualized in one of eight different ways according to the type of annotation, e.g., DAG for GO. Separately, the annotation can be analysed using statistical tests to identify over- or under-represented categories in the specified set as compared to a reference set. GOClust is a Perl program used to identify proteins from a list of proteins that are annotated to a selected GO term or its progeny terms [7,13]. Interestingly, all of the tools described above incorporate GO annotation to find commonalities within a list of proteins, emphasizing the importance of using GO annotation for analysing sets of molecules. Yet none of these tools provide an integrated display of results facilitating interpretation of the biological meaning of the protein set annotation.

Clustering proteins according to shared annotation may reveal related subsets that warrant further investigation. Two separate groups have clustered proteins by their annotation in order to identify incorrect annotations in curated databases. Kaplan and Linial measured the distance between any two proteins as a function of the number of terms that are annotated to both proteins, where less common terms, such as heat shock protein, score higher than more common terms, such as enzyme [14]. They identified successful hierarchical clustering as the point in the hierarchy at which one of the clusters contains no false positive annotations. The similarity score used by Kunin and Ouzounis incorporated the ratio of common to unique terms between the annotation of two SwissProt proteins and the frequency of those terms within SwissProt as a whole [15]. All proteins in SwissProt were then clustered into >43,000 clusters. Sequence similarity between proteins within clusters was found to be consistent overall, apart from six types of exceptions, one of which was SwissProt annotation errors.

As a first step towards investigating the feasibility of clustering proteins by annotation for the purpose of facilitating interpretation of HTP results, we have employed a graph similarity distance measure implemented in Bioconductor [16,17] and Partitioning Around Medoids (PAM) clustering to examine the annotation of two published HTP proteomic data sets. Zhu *et al*. [18], hereafter referred to as the Snyder data set, demonstrated that purified proteins representing most of the yeast proteome could be immobilized on chips and tested for interaction with proteins or lipids. The primary purpose of the publication was to demonstrate that the proteome array is able to detect known interactions in addition to identifying new ones, lending support to the usefulness of the technique. In Huang *et al*. [19], hereafter referred to as the Schreiber data set, proteins from the yeast proteome array that interacted with two small molecules of interest were tested using *in vivo *experiments to further examine whether the loss of the protein affected the cellular response to the presence of the inhibitors. From this, only one of 38 interacting proteins identified was chosen for further study. Here we have assembled these two sets of proteins as identified in screens of purified protein arrays and re-analysed them by clustering the proteins according to their GO annotation, thus generating new hypotheses about how proteins in these sets may function within the cell.

### Methodologies and concepts

#### Distance metric

**Figure 1:**
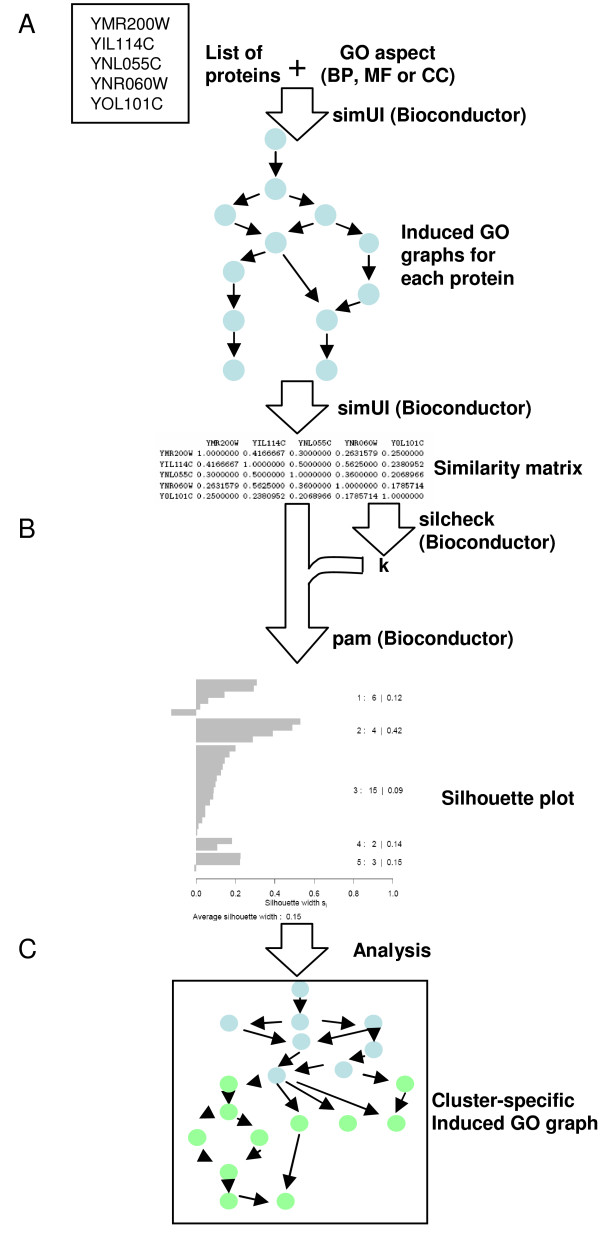
**Methodology for clustering a list of proteins by graph similarity of Gene Ontology annotation**. (A) The input to the methodology consists of a list of proteins and selection of one aspect of the Gene Ontology, i.e., Biological Process (BP), Molecular Function (MF) or Cellular Component (CC). The Bioconductor method *simUI *is then employed to generate a matrix of graph similarities between each pair of proteins in the list. (B) The Bioconductor method *silcheck *uses the similarity matrix to select the number of clusters, k. The Bioconductor method *pam *uses the similarity matrix and k to cluster the proteins. (C) The clustering result is then examined in further detail to produce a biological interpretation of the GO annotation of the inputted list of proteins.

**Figure 2:**
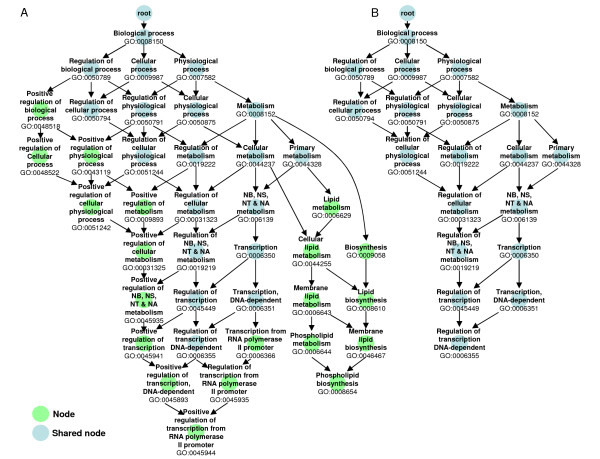
**Graph similarity scoring method**. The induced GO graphs for two yeast proteins illustrate graph similarity scoring using the Bioconductor method *simUI*. (A) The GO terms GO:0045944 positive regulation of transcription from RNA polymerase II promoter and GO:0008654 phospholipid biosynthesis are assigned to INO4/YOL108C. (B) The GO term GO:0006355 regulation of transcription, DNA-dependent is assigned to RSC30/YHR056C. The graph similarity between these two proteins is calculated by dividing the number of terms that are found in both of the individual induced GO graphs for each protein (shared nodes in blue) by the number of unique terms in both graphs. The graph similarity equals 20 shared nodes/40 unique nodes = 0.5

Our objective is to find clusters of proteins such that the proteins within a cluster are close from a biological perspective and correspondingly far from the proteins in other clusters. The biological perspective that we choose is GO annotation, and we define distance by referring to the GO graph. Of the many possible GO-based distance metrics (e.g. [20]), we choose the *simUI *metric included in the *GOstats *package of Bioconductor (Figure 1A), in part because of its universal availability. This metric is based on the notion of an induced GO graph and treats each of the three GO aspects separately (Biological Process (BP), Molecular Function (MF), Cellular Component (CC)). The GO terms to which a protein maps constitute the leaves of the graph; thus, a protein with more than one GO annotation will have more than one leaf in the induced GO graph. The complete induced GO graph consists of those leaves and all parents of those terms, and so on until the root node has been obtained. The graph similarity between two proteins calculated using *simUI *equals the number of common nodes in the two induced GO graphs divided by the number of nodes in the union of the two graphs, thus the similarity lies between 0 and 1. The associated dissimilarity is 1 – similarity. Figure 2 illustrates the *simUI *distance between two yeast proteins. Figure 2A shows the GO BP annotation for INO4/YOL108C (GO:0045944 positive regulation of transcription from RNA polymerase II promoter, GO:0008654 phospholipid biosynthesis) and Figure 2B shows the GO BP annotation for RSC30/YHR056C (GO:0006355 regulation of transcription, DNA dependent) as retrieved using GO version 1.10.0 in Bioconductor. The 20 GO terms found in both induced GO graphs are highlighted in blue and there are 40 unique GO terms in total. Therefore, the graph similarity between INO4/YOL108C and RSC30/YHR056C in GO BP is 20 common nodes/40 unique nodes = 0.5.

#### Clustering method and selecting k

The clustering method employed is Partitioning Around Medoids (PAM) (Figure 1B). The medoid of a cluster is the protein with smallest average dissimilarity to all other objects in the cluster. It is important to note that the medoid is an actual protein as opposed to an abstract entity such as the cluster mean and thus we find this feature is helpful in describing the clusters. For a specified number of clusters k, PAM begins by arbitrarily selecting k proteins to be medoids. It then forms clusters by grouping each protein with the closest medoid. The medoids are then recalculated and the proteins regrouped, and so on until the clusters cease to change.

The number of clusters, k, must be specified in advance of the clustering step. A review of 30 procedures for estimating k is given by Milligan and Cooper (1985) [21]. Dudoid and Fridlyand (2002) discuss several methods in the context of gene expression data [22]. Unfortunately, none of the available methods are completely satisfactory and there is no consensus about the choice of method. We have adopted a very common method that selects k to maximize the average silhouette (described below). This method was chosen because it utilizes the same framework employed in interpreting the clusters.

Cluster analysis is a descriptive technique that can reveal associations that may not be noticed otherwise. A larger value of k produces smaller clusters; a small cluster may be less informative in that it forms connections between fewer proteins and thus is less likely to point out novel associations. A small value of k can produce large clusters which may display associations that do not really exist in the underlying biology. Therefore, we have followed the default settings in the *silcheck *method in Bioconductor and limited the maximum number of clusters to 9. Additional subdivision of clusters can be based on biological knowledge or supplementary analysis, such as inspecting inter-protein GO distances or further cluster analysis within a protein cluster. Many of the methods for estimating k and cluster reproducibility depend on some form of resampling, such as resampling expression arrays, and are not applicable in the context of GO similarity.

#### Silhouette widths and silhouette plots

We assess our clustering results using the silhouette plot, which graphically illustrates the strength of the clustering for the entire data set, of each cluster and of the association of each protein to the cluster to which it is assigned (Figure 1B). We label each cluster in the silhouette plot with the GO annotation of the protein selected as the medoid to provide a first glance at the annotation patterns uncovered by clustering.

There are three types of silhouette widths found on a silhouette plot. The silhouette width for each object (e.g., protein) in the data set (s_i_) measures how well the object fits in the cluster to which it was assigned. For each object i, a_i _= average dissimilarity between i and all other objects of the cluster to which i belongs. Thus, if there is only one object in a cluster, s_i _= 0 without further calculation. For all other clusters C (i.e. all clusters other than the cluster to which i belongs), d_i_^C ^= average dissimilarity of i to all observations in C. Then b_i _= the smallest value of d_i_^C ^and thus represents the dissimilarity between i and its neighbour cluster, the nearest cluster to which i does not belong. Finally,

si=(bi−ai)max⁡(ai,bi)
 MathType@MTEF@5@5@+=feaafiart1ev1aaatCvAUfKttLearuWrP9MDH5MBPbIqV92AaeXatLxBI9gBaebbnrfifHhDYfgasaacH8akY=wiFfYdH8Gipec8Eeeu0xXdbba9frFj0=OqFfea0dXdd9vqai=hGuQ8kuc9pgc9s8qqaq=dirpe0xb9q8qiLsFr0=vr0=vr0dc8meaabaqaciaacaGaaeqabaqabeGadaaakeaacqqGZbWCdaWgaaWcbaGaeeyAaKgabeaakiabg2da9maalaaabaGaeiikaGIaeeOyai2aaSbaaSqaaiabbMgaPbqabaGccqGHsislcqqGHbqydaWgaaWcbaGaeeyAaKgabeaakiabcMcaPaqaaiGbc2gaTjabcggaHjabcIha4jabcIcaOiabbggaHnaaBaaaleaacqqGPbqAaeqaaOGaeiilaWIaeeOyai2aaSbaaSqaaiabbMgaPbqabaGccqGGPaqkaaaaaa@4579@

The average s_i _of each cluster (s_i_^C^) is the mean of the s_i _values for all objects in the cluster. The average s_i _for the entire data set (s_i_^D^) is the mean of the s_i _values for all objects in the set. In general, the clustering results for the object, cluster or data set are strongest when the s_i_, s_i_^C ^or s_i_^D ^are close to 1 (Table 1).

More specifically, when s_i _is close to 1, the average dissimilarity of this object to other objects in its cluster is much smaller than the average dissimilarity of this object to the objects in the neighbouring cluster [23]. Therefore, this object appears to be assigned to the correct cluster. When s_i _is close to 0, the object lies equally far away from its own cluster and its closest neighbouring cluster [23], hence this is more likely to be an intermediate object that lies between two clusters. Alternatively, the object may legitimately belong to both clusters. An s_i _that is close to -1 indicates that the object may have been misclassified as it is much closer to the objects in another cluster than to those in its own cluster [23].

Similarly, an s_i_^C ^close to 1 indicates that all of the objects in that cluster are very similar whereas an s_i_^C ^below 0.25 indicates that this cluster is not clearly separated from the other cluster(s) [23].

Kaufman and Rousseeuw describe a subjective interpretation of s_i_^D ^based solely on experience [23]. They find that an s_i_^D ^of 0.51–1.0 indicates a reasonable to strong clustering structure has been found. An s_i_^D ^of 0.26–0.50 indicates a weak clustering structure that could be artificial and the use of additional methods of data analysis is recommended. An s_i_^D ^below 0.25 indicates that no substantial structure has been found [23]. As this is by the authors own admission a subjective interpretation, average silhouette widths that fall below 0.25 do not always produce meaningless clustering results.

#### Interpretation and evaluation of clusters

We evaluate our clustering results by examining the induced GO graph of the proteins in a given cluster (Figure 1C). At this stage, scientific knowledge of the original purpose of the screen and the molecules being studied allows the assessment of whether the clustering procedure described above reveals interesting associations in cellular role, molecular function or localization for further experimentation and study. The interpretation of the clusters may lead to a revision of the number of clusters and reiteration of the cluster analysis step. Due to the inherently close relationships between biological annotation terms and the interconnectedness within each aspect of the GO, one would not expect clustering by GO annotation to produce strong clustering structures with clear delineations between clusters. Therefore, we expect that any clusters identified will require further analysis using complementary methods such as examination of other cluster properties or detailed examination of the proteins in the cluster.

To validate the methodology of clustering by annotation, we compare our clustering results with an analytic approach commonly used in DNA microarray analysis, identification of statistically over-represented GO terms [24]. We test the appropriateness of our medoid labels by determining whether the GO annotation of the medoid protein is a statistically enriched GO term for that cluster. That is, if the GO annotation of the medoid protein is a statistically enriched GO term for the proteins in that cluster, it indicates that the medoid label accurately represents the GO annotation of the proteins in that cluster.

In a separate but related approach, we test whether clustering of proteins by annotation is able to identify novel annotation patterns within the data set by comparing the GO terms of the medoid proteins to the statistically enriched GO terms for the entire data set. If the medoid GO terms are statistically enriched terms in the entire data set, then our approach has not provided any new information about this data set. If, however, the medoid GO terms are not statistically enriched in the entire data set, our approach has revealed novel annotation patterns within the set of molecules that would not have been identified otherwise.

## Results

### Generation of similarity matrices for sample data sets

Most of the proteins in the two sample data sets (see Methods) had corresponding Entrez Gene identifiers. Specifically, 37 of the 39 proteins in the Schreiber data set and 91 of the 99 proteins in the Snyder data set had Gene IDs. Similarity scores were calculated using Bioconductor *simUI *for each data set (Schreiber, Snyder) for each GO aspect (BP, MF and CC), which generated six sets of similarity scores.

### Schreiber data: proteins that bind small molecule inhibitors of rapamycin can play a role in transport or redox reactions or be found in nuclear sub-complexes

**Figure 3:**
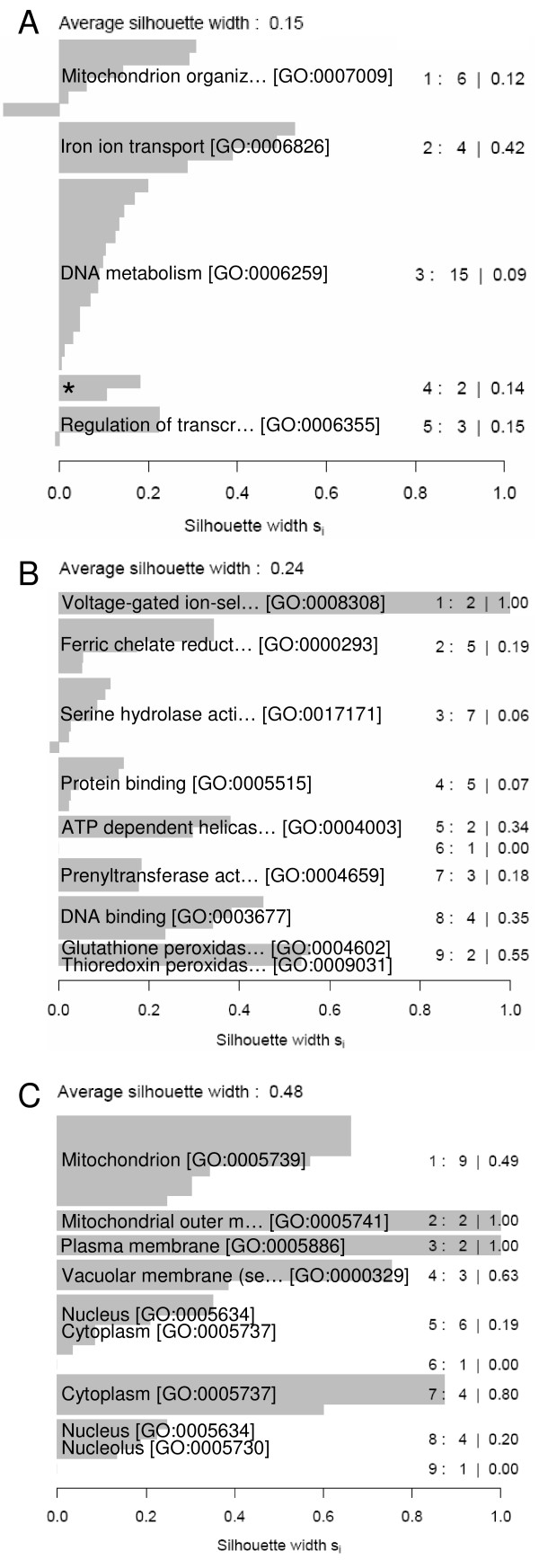
**Silhouette plots of PAM clustering results for Schreiber data set**. Silhouette plots of PAM clustering results for 37 rapamycin-inhibitor binding proteins for GO (A) BP, (B) MF and (C) CC. Proteins assigned either the unknown term from each GO aspect (GO:0000004 biological process unknown, GO:0005554 molecular function unknown and GO:0008372 cellular component unknown) or using the evidence code Inferred from Electronic Annotation were not included in the clustering. Therefore 30 proteins were clustered in BP, 31 in MF and 32 in CC. The silhouette width for the entire set (average silhouette width, s_i_^D^) is found at the top of each figure whereas the silhouette width for each cluster (s_i_^C^) is found on the right-hand side of the figure with the cluster number (left of the colon) and number of proteins in each cluster (right of the colon). Each cluster is labelled with the GO annotation of the medoid, except BP cluster 4 as the text did not fit on the figure. Each protein is represented by a bar and the width of the each bar represents the silhouette width for each protein (s_i_). * GO annotation for BP cluster 4 is GO:0046856 phosphoinositide dephosphorylation, GO:0048017 inositol lipid-mediated signaling and GO:0030476 spore wall assembly (sensu Fungi).

Screening the yeast proteome array with two different small molecules, SMIR3 and SMIR4 that were able to inhibit rapamycin, isolated 39 SMIR-binding proteins [19], of which 37 were analysed here. For the Schreiber data set BP k = 5 was selected and for MF and CC k = 9 was selected. PAM clustering was performed for the three sets of dissimilarity scores (Figure 3A–C) [see Additional file 1].

The s_i_^D ^for each of the three data sets is shown at the top of each figure. Each cluster is labelled with the GO annotation(s) assigned to the medoid and the s_i_^C ^(Figure 3A–C). The clustering structures in BP and MF were weak overall (BP s_i_^D ^= 0.15, MF s_i_^D ^= 0.24) and strong in CC (s_i_^D ^= 0.48). Upon further analysis, some of the clusters within each of the GO aspects were informative.

**Figure 4:**
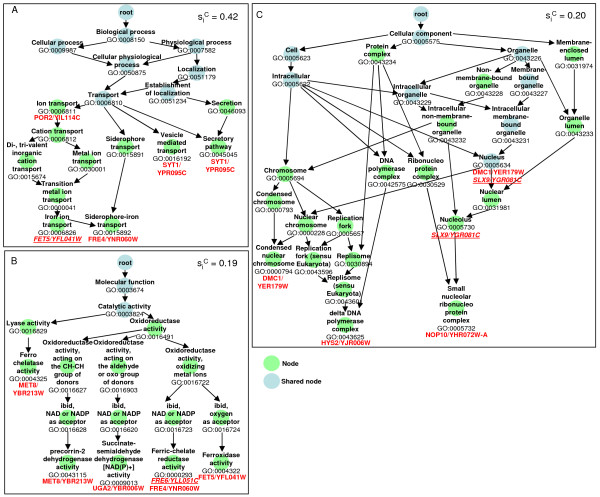
**Induced GO graphs for one cluster from each GO aspect for the Schreiber data set**. Induced GO graphs containing the BP, MF or CC annotation for the proteins found in Schreiber (A) BP cluster 2, (B) MF cluster 2, and (C) CC cluster 8, respectively. Nodes found in all of the individual induced GO graphs for the proteins in the cluster are shown in blue. The silhouette width for each cluster (s_i_^C^) is shown in the upper right hand corner. The medoid protein for each cluster is italicized and underlined.

The Schreiber BP data set (Figure 3A) had a weak s_i_^D ^as mentioned above, as did 4 of the 5 s_i_^C ^values. However, Schreiber BP cluster 2 had a strong s_i_^C ^of 0.42. We collected the GO annotation for the four proteins in this cluster for further examination (Figure 4A). All four proteins are involved in transport.

The Schreiber MF data set (Figure 3B) had a s_i_^D ^just below 0.25 which suggests that no substantial clustering structure was found. Indeed, MF clusters 3 and 4 had low s_i_^C ^values indicating that these clusters were not very clearly separated from other clusters. MF cluster 6 had s_i_^C ^= 0 because there was only one protein in the cluster. The two proteins in MF cluster 1 (POR1/YNL055C, POR2/YIL114C) were both assigned to the GO:0008308 voltage-gated ion-selective channel activity and were thus a perfect cluster (s_i_^C ^= 1.00). MF clusters 5, 8 and 9 were also small clusters containing 2, 4 and 2 proteins with s_i_^C ^values of 0.34, 0.35 and 0.55, respectively. Upon examination, it was clear that the GO MF annotations for the proteins in each of these clusters are closely related. MF clusters 2 and 7 had moderate s_i_^C ^values (0.19 and 0.18) and thus may reveal novel associations between proteins in this set that may not have been readily observed. We examined the GO annotation of the five proteins in MF cluster 2 in detail (Figure 4B) and found that all of these proteins are able to catalyze redox reactions. Three of the five proteins use iron as the electron donor while a fourth chelates iron. This may reveal a novel affinity of SMIR3 and SMIR4 for proteins that interact with double-charged iron (Fe^2+^).

The Schreiber CC data set (Figure 3C) had a strong s_i_^D ^value (0.48). In fact, CC clusters 2 and 3 had perfect s_i_^C ^values, meaning that the proteins in these clusters have identical GO CC annotation, while CC clusters 1, 4 and 7 also had high s_i_^C ^values (0.49, 0.63 and 0.80). We would expect that the 9, 3 and 4 proteins in these clusters, respectively, would have very similar cellular localization annotation. CC clusters 6 and 9 had s_i_^C ^= 0 as both clusters contained only one protein. We chose to examine CC cluster 8 in more detail as it had a moderate s_i_^C ^value (0.20) (Figure 4C). The GO subgraph reveals that all four proteins are found within the nucleus, thus the clusters labels from the medoid of GO:0005634 nucleus and GO:0005730 nucleolus are apt. However, there is quite specific knowledge about the complexes within the nucleus in which three of these proteins are found. As a result, the GO graph contained many detailed GO CC terms causing the graph similarity between these proteins and the corresponding s_i_^C ^to appear lower than it might otherwise appear. This cluster, along with MF cluster 2, demonstrates that although experience using PAM indicates that clusters with s_i_^C ^<= 0.25 may not be interpretable, this does not hold true in all cases and protein clusters with moderate s_i_^C ^values should be considered for biological interpretation.

### Snyder data: proteins that bind phosphoinositides can be related to transport, transfer of phosphorous-containing groups or intracellular organelles

**Figure 5:**
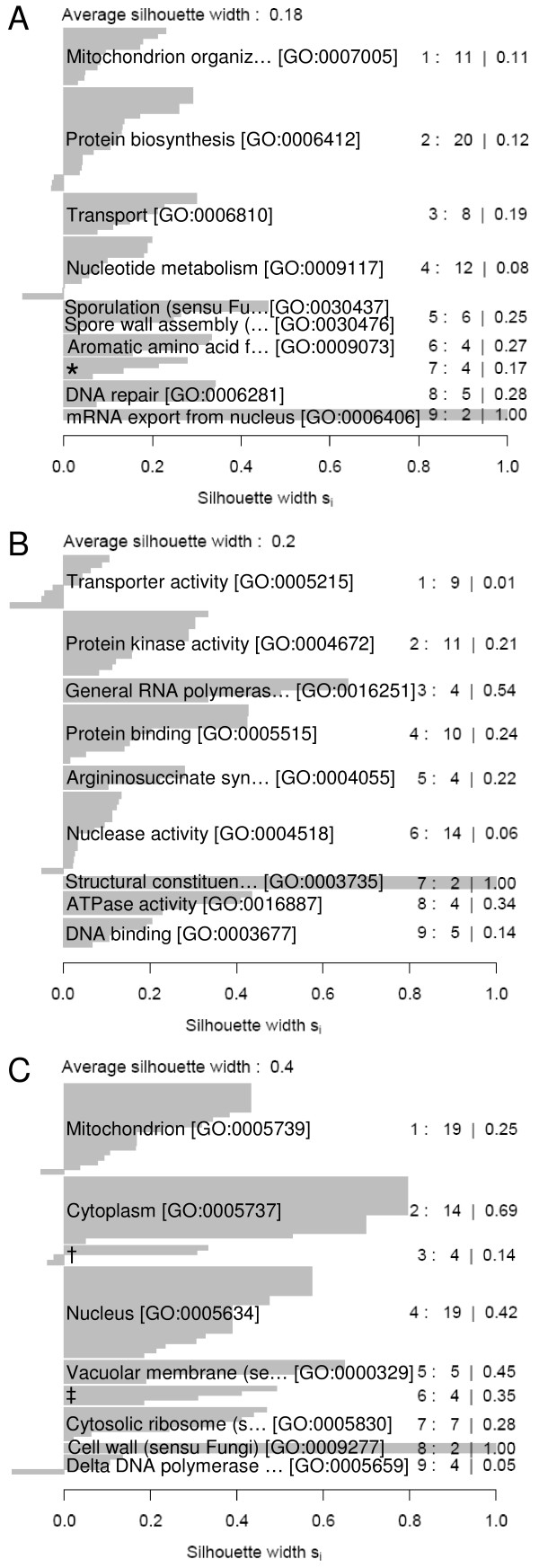
**Silhouette plots of PAM clustering results for Snyder data set**. Silhouette plots of PAM clustering results for 91 phospholipid binding proteins for GO (A) BP, (B) MF and (C) CC. Proteins assigned either the unknown term from each GO aspect (GO:0000004 biological process unknown, GO:0005554 molecular function unknown and GO:0008372 cellular component unknown) or using the evidence code Inferred from Electronic Annotation were not included in the clustering. Therefore 72 proteins were clustered in BP, 63 in MF and 78 in CC. The silhouette width for the entire set (average silhouette width, s_i_^D^) is found at the top of each figure whereas the silhouette width for each cluster (s_i_^C^) is found on the right-hand side of the figure with the cluster number (left of the colon) and number of proteins in each cluster (right of the colon). Each cluster is labelled with the GO annotation of the medoid, except BP cluster 7 and CC clusters 3 and 6 as the text did not fit on the figure. Each protein is represented by a bar and the width of the each bar represents the silhouette width for each protein (s_i_). * GO annotation for BP cluster 7 is chromatin silencing [GO:0006342] and histone deacetylation [GO:0016575]. † GO annotation for CC cluster 3 is mitochondrial inner membrane [GO:0005743], integral to membrane [GO:0016021] and mitochondrial nucleoid [GO:0042645]. ‡ GO annotation for CC cluster 6 is plasma membrane [GO:0005886] and integral to membrane [GO:0016021].

Testing purified yeast proteins on an array for interaction with several phosphoinositides identified a set of 99 proteins [18], of which 91 were analysed here. For the Snyder data set, k = 9 was selected for all three GO aspects. Silhouette plots of the PAM clustering results for each GO aspect are labelled with s_i_^D ^at the top of each figure and medoid GO annotation, cluster number, number of proteins in the cluster and s_i_^C ^for each cluster (Figure 5A–C) [see Additional file 2]. The medoid labels for BP cluster 7 (Figure 5A) and CC clusters 3 and 6 (Figure 5C) are listed in the figure legend. Overall, the s_i_^D ^for BP and MF were low (BP s_i_^D ^= 0.18, MF s_i_^D ^= 0.20) while CC was moderate (s_i_^D ^= 0.40) indicating that further analysis is required.

**Figure 6:**
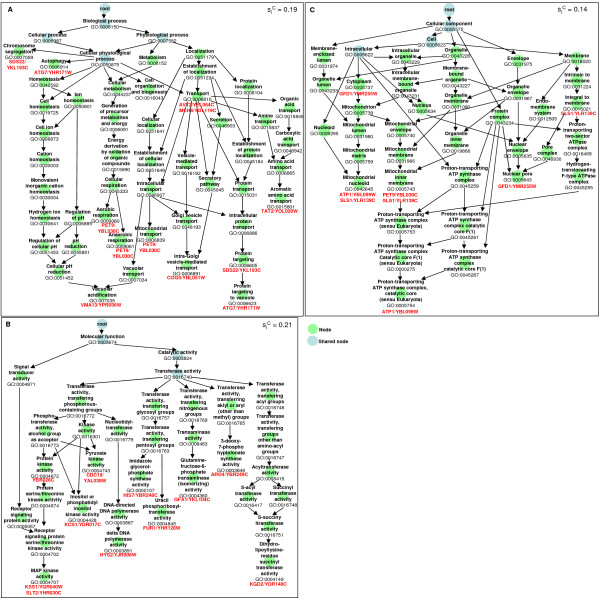
**Induced GO graphs for one cluster from each GO aspect for the Snyder data set**. Induced GO graphs containing the BP, MF or CC annotation for the proteins found in Snyder (A) BP cluster 3, (B) MF cluster 2, and (C) CC cluster 3, respectively. Nodes found in all of the individual induced GO graphs for the proteins in the cluster are shown in blue. The silhouette width for each cluster (s_i_^C^) is shown in the upper right hand corner. The medoid protein for each cluster is italicized and underlined.

Snyder BP clusters 1, 2 and 4 had low s_i_^C ^values (0.11, 0.12 and 0.08 respectively) (Figure 5A). BP cluster 9 contained two proteins and had a perfect s_i_^C ^(1.00). The remaining BP clusters (3, 5, 6, 7 and 8) had s_i_^C ^values between 0.17 and 0.28 suggesting that they should be examined further. These clusters contain between 4 and 8 proteins; we selected the largest cluster, BP cluster 3, for further analysis (Figure 6A). The graph of the GO BP annotation of these eight proteins appears to uncover widely varying cellular roles but on closer examination we see that seven of the eight proteins are involved in transport. Indeed, five are annotated to progeny terms of GO:0046907 intracellular transport. It is the additional GO BP annotations associated with some of the proteins in this subset, i.e. GO:0007059 chromosome segregation, GO:0006914 autophagy, GO:0009060 aerobic respiration and GO:0009061 anaerobic respiration, that make the GO graph appear complex.

Snyder MF cluster 1 contained nine proteins whose s_i _values range from 0.11 to -0.12 and thus had a s_i_^C ^value close to 0 (Figure 5B). MF cluster 6 contained 14 proteins and also had a very low s_i_^C ^value (0.06) but most of these proteins had s_i _values above 0. Snyder MF cluster 7 had a perfect s_i_^C ^value and consisted of two proteins annotated to GO:0003735 structural constituent of ribosome. MF clusters 3 and 8 had moderate to high s_i_^C ^values (0.54 and 0.34 respectively). Inspection of the GO annotation of the proteins in each of these clusters revealed subsets of proteins with very closely related GO MF annotation (RNA polymerase II transcription factor and nucleotide phosphatase activity, respectively). MF clusters 2, 4, 5 and 9 had s_i_^C ^values ranging from 0.14 to 0.24 and contain between 4 and 11 proteins. We again chose the largest cluster, MF cluster 2, to examine in detail (Figure 6B). The GO graph shows that all eleven proteins are enzymes belonging to EC class 2, transferases. Although the molecules that these enzymes transfer vary from glycosyl to nitrogenous to acyl, there is a subset of six proteins that transfer phosphorous-containing groups.

Many of the clusters in the Snyder CC clustering result (Figure 5C) had high s_i_^C ^values indicating tight clusters. Specifically, the s_i_^C ^values for CC clusters 1, 2, 4, 5, 6, 7 and 8 ranged from 0.25 to 1.00. Examination of the GO annotation of the proteins in these clusters quickly revealed that the medoid GO annotation is an accurate and useful representation. CC cluster 9 had a very low s_i_^C ^value of 0.05 but, as was the case with Schreiber CC cluster 8, the detailed biological knowledge that exists about nuclear sub-complexes allows the construction of a more detailed GO tree for these terms, which then lowered the apparent similarity between these proteins. All four proteins in Snyder CC cluster 9 are found in nuclear complexes and three of four are known to associate with chromosomes. We chose to illustrate examination of the GO annotation with the four proteins in CC cluster 3 (s_i_^C ^= 0.14) (Figure 6C). Three of these proteins are found in the mitochondrion, either in the mitochondrial nucleoid or mitochondrial inner membrane. The fourth protein is found in both the cytoplasm and nuclear pore complex. It is not surprising to see that GFD1/YMR255W, which is found in the cytoplasm and nuclear pore, has a negative s_i _value for its assignment to this cluster (-0.02) as there are few shared ancestor terms between these two terms and the mitochondrion-related terms. However, it is surprising that PET9/YBL030C has a negative s_i _value for its assignment to this cluster as it is assigned to the same GO term as one of the other proteins in this cluster, SLS1/YLR129C (GO:0005743 mitochondrial inner membrane). We might expect the *simUI *similarity between these two proteins to be high since they are assigned to the same GO term but SLS1/YLR129C is also assigned to two other GO CC terms, GO:0042645 mitochondrial nucleoid and GO:0016021 integral to membrane. The induced GO graph for SLS1/YLR129C therefore contained many nodes that were not found in the induced GO graph for PET9/YBL030C, thus reducing their *simUI*-calculated graph similarity.

### GO annotation of medoid is often a statistically enriched GO term for the corresponding cluster

We investigated whether the GO annotations of the proteins selected as the medoids, which are used as cluster labels, are actually representative of the annotation of the proteins assigned to the cluster by comparing the medoid GO terms to the statistically enriched GO terms for each cluster. Identifying statistically enriched GO terms for a set of molecules is a common method of analysis for microarray results [24-26]. In this method, the GO annotation of a selected subset of molecules is compared to the GO annotation of a reference set of molecules (e.g. the yeast proteome or all molecules on an array) and any term or any of its ancestor terms that occur more often in the selected subset than in the reference set are said to be statistically enriched. If the GO annotation of the medoid is representative of the annotation of the proteins in the cluster, we would expect the cluster label GO terms or closely related GO terms to be statistically enriched for that cluster.

We employed FunSpec, a free online tool that identifies statistically enriched annotation for yeast molecules via hypergeometric distribution [25]. First we submitted the list of molecules for each of the 50 clusters (Schreiber: 5 BP, 9 MF, 9 CC, Snyder: 9 BP, 9 MF, 9 CC) to FunSpec [27] and collected the statistically enriched GO terms from the relevant GO aspect (i.e., BP terms only for BP clusters, *etc*). We found that 42 of 50 clusters had one or more statistically enriched GO terms (p < 0.01). For 33 of 42 clusters, the medoid GO term chosen as the cluster label was (one of) the statistically enriched GO term(s). For 8 of the 9 remaining clusters, one or more of the statistically enriched GO terms was an ancestor or progeny term of the medoid GO term, indicating that the medoid GO term is related to the statistically enriched GO terms. Indeed, 4 of 8 related terms were direct parent terms of the medoid GO term. In summary, 78% (33/42) of the cluster labels selected by PAM are statistically enriched GO terms for their cluster and are thus appropriate and useful GO terms to apply as cluster labels.

### GO annotation of medoid proteins uncover patterns not found in statistically enriched GO terms for the data set

We then examined whether the approach of clustering proteins by their annotation revealed patterns in the protein set that were not revealed by existing methods. As mentioned, sets of genes identified in DNA microarray experiments are often examined for statistically over-represented GO terms. We hypothesized that if the clustering is able to uncover new annotation patterns, the GO terms assigned to the medoid proteins or closely related GO terms would be distinct from the list of GO terms over-represented in the entire data set.

We submitted six lists of proteins representing the protein sets that were clustered for each of the two data sets for each of the three GO aspects. The number of proteins was slightly different for each GO aspect from the same dataset because proteins that were annotated to unknown GO terms (GO:0000004 biological process unknown, GO:0005554 molecular function unknown and GO:0008372 cellular component unknown) were excluded. Thus, the following six sets were submitted to FunSpec: (1) Schreiber BP – 30 proteins, (2) Schreiber MF – 31 proteins, (3) Schreiber CC – 32 proteins, (4) Snyder BP – 72 proteins, (5) Snyder MF – 63 proteins and (6) Snyder CC – 78 proteins. We searched the list of statistically enriched GO terms for each of these six data sets for the GO terms assigned to the medoid proteins. Only 8 of the 50 medoid GO terms were found to also be statistically enriched when examining the entire data set. Even if we expanded the search to look for any ancestors or progeny of the medoid GO terms, only 19 of 50 cluster labels (38%) are identified by FunSpec as statistically over-represented. This suggests that the process of clustering the proteins by their GO annotation and selection of a representative GO term with which to label each cluster is a valuable and useful way to identify novel annotation patterns within the data set that are not identified by existing methods.

## Discussion

Compared to listing the statistically enriched GO terms for a set of molecules, this novel approach generates sets of related molecules labelled with representative GO terms that are in fact assigned to one of the molecules in the set. The GO annotations of the medoid proteins help to uncover patterns that are not identified by searching for statistically enriched GO terms. These labelled subsets make the annotation patterns found within the set of molecules much more readily apparent than annotation patterns represented in a DAG.

We examined a set of proteins identified by their ability to interact with one of two small molecule inhibitors of rapamycin [19]. One subset was found to contain four proteins involved in transport. It is known that mammalian target of rapamycin (mTOR) is involved in nutrient and protein transport [28,29] and that rapamycin inhibits this function of the mTOR pathway. Thus it is reasonable that small molecules that inhibit the effect of rapamycin on the cell could also affect transport pathways. Another subset contained four proteins that bind Fe^2+^. It has been shown that removal of growth factors can cause a loss of surface transporters for several types of molecules, including iron [30]. By exogenously stimulating growth factor receptor pathways, an mTOR-dependent mechanism can maintain these transporters on the cell surface. Thus, it would be interesting to investigate whether the subset of proteins identified here are involved in the regulation of cell surface iron transporters by the mTOR pathway. A final subset contains four proteins known to be located in nuclear complexes. Study of these proteins may reveal the mechanisms by which the mTOR pathway is involved in various nuclear events such as DNA damage and transcription [29].

We also examined a set of proteins known to interact with phospholipids [18]. We identified a subset of seven proteins that are involved in different types of transport. Many of the processes involve intracellular membrane-bound compartments such as Golgi apparatus, mitochondria and vacuole. Thus, it is reasonable to expect that these proteins bind phospholipids in the membranes of these compartments. We also identified a group of eleven transferases, of which six transfer phosphorous-containing groups. Many protein kinases that are involved in signal transduction are known to bind and be regulated by phospholipids [31,32]. Thus it would be interesting to investigate whether the activity of these kinases are regulated by their interaction with phospholipids. Lastly, we identified a cluster of four proteins with phospholipid-binding ability that are localized to the mitochondrial inner membrane or the membrane-integral nuclear pore complex, both of which are phospholipid-containing structures. Examination of these proteins may determine whether their localization depends on their ability to bind phospholipids.

For both of these data sets, our analysis revealed annotation patterns that were not identified by the authors in the original article nor were they identified by an existing method for analysing the annotation of sets of molecules. Indeed, the annotation patterns that were identified themselves suggest potential follow-up experiments to examine the mechanisms and impact of the interactions identified in the protein array screens.

We are currently working to create a web-based software tool to automate this method of analysis. In principle, this method does not only apply to protein array results but could also be used to analyse any set of genes or proteins. Preliminary work to analyse two protein datasets from higher organisms shows, not unexpectedly, that fewer of the proteins in the dataset have GO annotation than was found to be the case with the yeast datasets but that clusters maintain moderate to high s_i_^C ^values (0.18–0.41) (data not shown). While the annotation of proteins from higher organisms is not as comprehensive as the annotation of yeast proteins, we have found that analysis of the existing publicly available GO annotation still produces functional themes that suggest testable hypotheses. As annotation of higher organisms grows, the application of this analytic approach will improve.

## Conclusion

The growing field of high-throughput experimentation is creating a rising need for tools that facilitate the integrated analysis of sets of molecules. Clustering can be used to identify annotation patterns within a set of proteins, such as is generated by protein array screens. Visual display of these annotation patterns can suggest new testable hypotheses as the basis for further analysis.

## Methods

### Collection of sample data sets

The results of two yeast proteome array screens were selected for analysis. The set of 39 proteins that bind to either Small Molecule Inhibitor of Rapamycin (SMIR)3 or SMIR4 was obtained from Supplementary Table 3 of Huang *et al *[19] and is hereafter referred to as the Schreiber data set. The set of 99 proteins that bind to phosphatidylinositol-and phosphatidylcholine-containing liposomes but not liposomes containing only phosphatidylcholine was obtained from Supplementary Table 1 of Zhu *et al *[18] and is hereafter referred to as the Snyder data set. A Perl program taking systematic open reading frame (ORF) names for each protein in the data sets as input was used to obtain standard names and Entrez Gene identifiers (Gene IDs) from the file gene_info.gz [33]. Thus for each data set, a list of systematic ORF names, standard names and corresponding Gene IDs was generated. All proteins in the Schreiber data set are classified by SGD as having Feature Type of verified or uncharacterized. Most proteins in the Snyder data set are classified by SGD as having Feature Type of verified or uncharacterized except two pseudogenes (YCL075W and FDH2/YPL275W), one transposable element (YNL054W-A) and one silenced gene (HMRA1/YCR097W). These proteins were included in the cluster analysis but not the biological analysis.

### Distance measure by graph similarity

The distance between each pair of proteins within each data set was determined using GO version 1.10.0 in Bioconductor [16,17]. Gene IDs were used to retrieve three induced GO graphs for each protein, one for each branch of the Gene Ontology (GO), molecular function (MF), biological process (BP) and cellular component (CC). Note that proteins with unknown GO annotations (GO:0000004 biological process unknown, GO:0005554 molecular function unknown and GO:0008372 cellular component unknown) and GO annotations assigned using the evidence code Inferred from Electronic Annotation were excluded. The similarity between each pair of proteins within each branch of GO was then determined using the *simUI *method in Bioconductor. This measure of similarity between two proteins falls between 0 and 1, where 1 represents proteins that have identical GO annotation. Note that three similarity matrices corresponding to the three branches of the GO were generated for each of the Schreiber and Snyder data sets.

### Clustering and visualization

Because the selected clustering method, Partitioning Around Medoids (PAM), requires input of dissimilarity between objects, the similarity matrices were converted to dissimilarity matrices using the equation dissimilarity = 1-similarity. The method *silcheck *in Bioconductor was used to select the number of clusters, k, based on the maximum average silhouette. Bioconductor was also used to perform PAM clustering and generate silhouette plots. Induced GO graphs were created manually.

## Authors' contributions

CW conceived of the study, collected the sample data, participated in the statistical analysis and interpretation of results and drafted the manuscript. CJM participated in the design of the study and interpretation of results and helped to draft the manuscript. DT participated in the design of the study, performed the statistical analysis and helped to draft the manuscript. All authors read and approved the final manuscript.

## Supplementary Material

Additional File 1 **Supplementary Table 2. Clustering results for 37 rapamycin-inhibitor binding proteins**. For each protein in the Schreiber data set, this table identifies for each GO aspect (BP, MF, CC) (1) the cluster to which the protein was assigned and (2) the silhouette width of the protein for this cluster assignment. The cluster and silhouette width for the protein that was selected as the medoid for each cluster is shown in bold. Click here for file

Additional File 2 **Supplementary Table 3. Clustering results for 91 phospholipid binding proteins**. For each protein in the Snyder data set, this table identifies for each GO aspect (BP, MF, CC) (1) the cluster to which the protein was assigned and (2) the silhouette width of the protein for this cluster assignment. The cluster and silhouette width for the protein that was selected as the medoid for each cluster is shown in bold. Click here for file

## Figures and Tables

**Table 1:** Interpretation of PAM silhouette widths

Statistic	Value	Interpretation
s_i_	close to 1	object assigned to correct cluster
	close to 0	intermediate object between two clusters
	close to -1	misclassified object

s_i_^C^	close to 1	well separated cluster
	< 0.25	cluster not well separated from neighbour(s)

s_i_^D^	0.51–1.00	reasonable to strong clustering structure
	0.26–0.50	weak clustering structure, use additional methods
	0.00–0.25	no substantial clustering structure
